# Supplementary material for: The stabilized supralinear network accounts for the contrast dependence of visual cortical gamma oscillations
Source: PLoS Comput Biol. 2024 Jun 27;20(6):e1012190. doi: 10.1371/journal.pcbi.1012190 (PMC11236182; doi:10.1371/journal.pcbi.1012190)
Supplement: S1 Table — In Figs 2, 3 and 5, parameters were sampled independently and uniformly from the ranges given in the table, except for enforcing three inequality constraints (i.e., sampled parameter sets violating any of these inequalities were rejected). See the main text (Methods) for details. †: these were the ranges for sampled λEE and λIE of the columnar model; these parameters were zero in the non-columnar model. (PDF) [file pcbi.1012190.s001.pdf]

Parameter	Fig. 1	Figs. 2-3	Fig. 4	Fig. 5	Fig. 6	Unit	Description
n	2					-	power-law I/O exponent
k	0.0194					$mV^{-2} \cdot ms$	power-law I/O pre-factor
τ_{corr}	5					ms	noise correlation time
τ_{AMPA}	5					ms	AMPA decay time
τ_{GABA}	7					ms	$GABA_A$ decay time
τ_{NMDA}	100					ms	NMDA decay time
ρ_N	0.39	0 - 0.5	0.39	0.3 - 0.5	0.45	-	NMDA share of excitation
J_{EE}	124	100 - 300	124	100 - 300	165	mV	total E $\to$ E connection weight
J_{IE}	116	100 - 300	116	100 - 300	123	mV	total E $\to$ I connection weight
J_{EI}	103	50 - 150	103	50 - 150	114	mV	total I $\to$ E connection weight
J_{II}	59.3	50 - 150	59.3	50 - 150	57.1	mV	total I $\to$ I connection weight
g_E	21.9	10 - 30	21.9	10 - 30	21.7	mV/s	*E* feedforward current per 1% contrast
g_I	10.3	5 - 15	10.3	5 - 15	10.6	mV/s	*I* feedforward current per 1% contrast
λ_{EE}	-	-	0.72	0.25 - 0.75†	0	-	locality of E $\to$ E connections
λ_{IE}	-	-	0.70	0.25 - 0.75†	0	-	locality of E $\to$ I connections
σ_{EE}	-	-	0.296	0.15 - 0.60	0.265	mm	range of E $\to$ E connections
σ_{IE}	-	-	0.554	0.15 - 0.60	0.294	mm	range of E $\to$ I connections
σ_{EI}	-	-	0.09			mm	range of I $\to$ E connections
σ_{II}	-	-	0.09			mm	range of I $\to$ I connections
N_{col}	1	1	17^2			-	number of cortical columns
L	-	-	6.4			mm	retinotopic network width
Δx	-	-	0.4			mm	cortical column width
M	-	-	2			mm/degrees	cortical magnification factor
w_{RF}	-	-	0.04°			degrees	grating input's margin width
σ_{Gabor}	-	-	0.5°			degrees	Gabor stimulus sigma

S1 Table. Parameters of models used in different figures of the main text. In figures 2, 3, and 5, parameters were sampled independently and uniformly from the ranges given in the table, except for enforcing three inequality constraints (*i.e.*, sampled parameter sets violating any of these inequalities were rejected). See the main text (Methods) for details. †: these were the ranges for sampled λ_{EE} and λ_{IE} of the columnar model; these parameters were zero in the non-columnar model.
