# Supplementary figures and images for: The stabilized supralinear network accounts for the contrast dependence of visual cortical gamma oscillations
Source: PLoS Comput Biol. 2024 Jun 27;20(6):e1012190. doi: 10.1371/journal.pcbi.1012190 (PMC11236182; doi:10.1371/journal.pcbi.1012190)

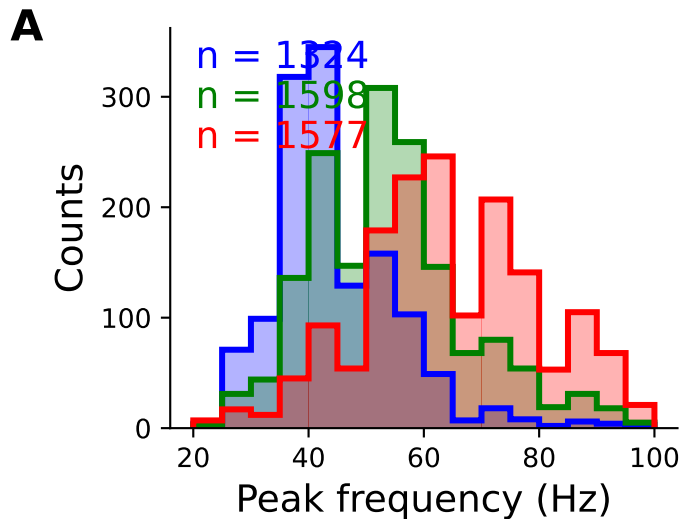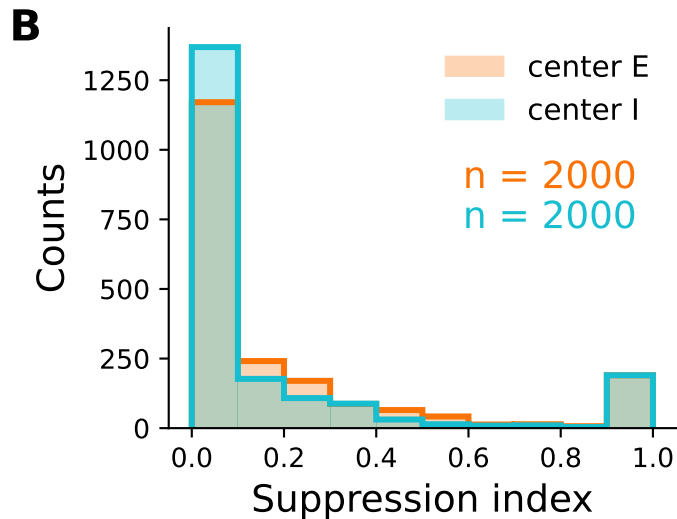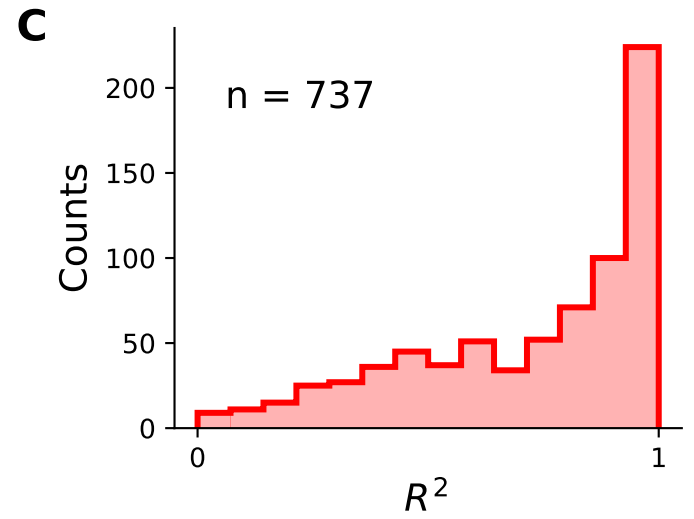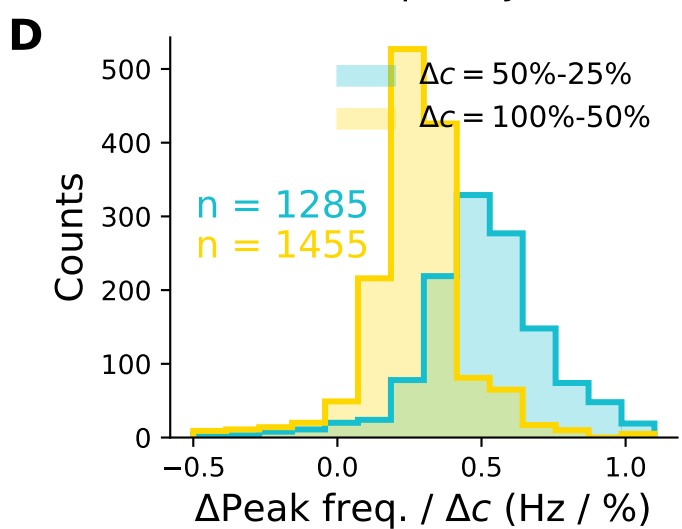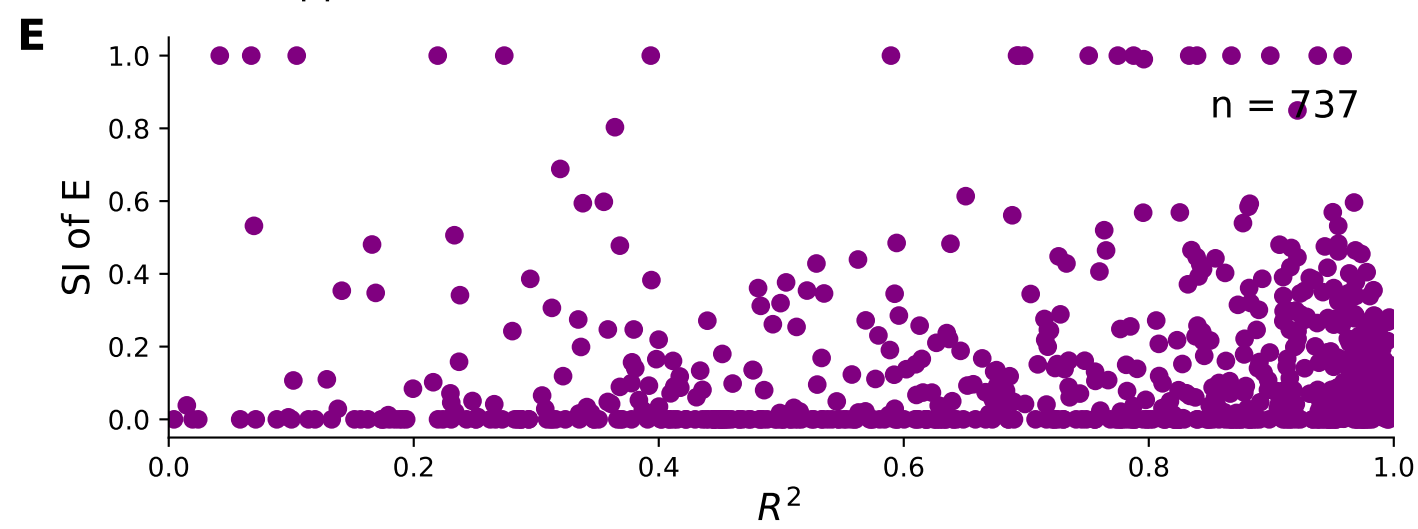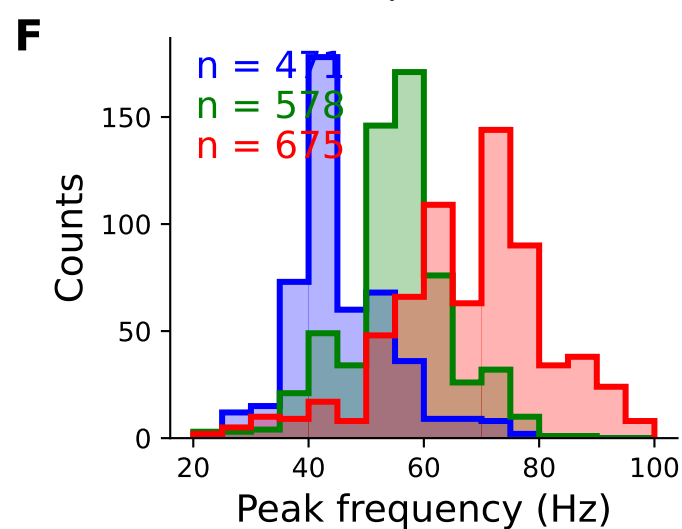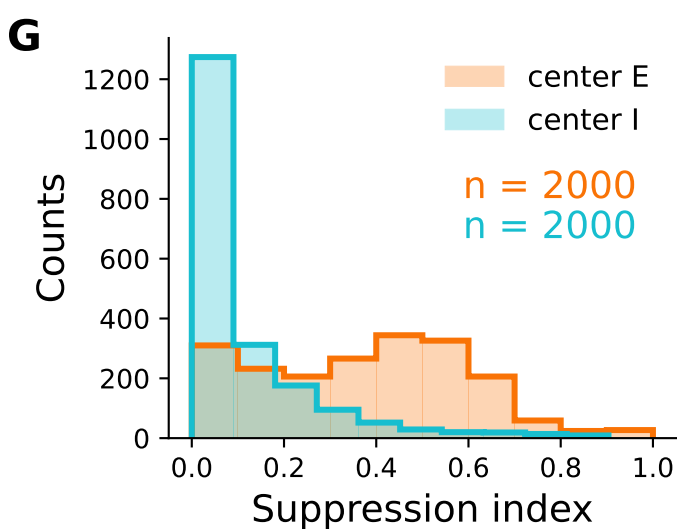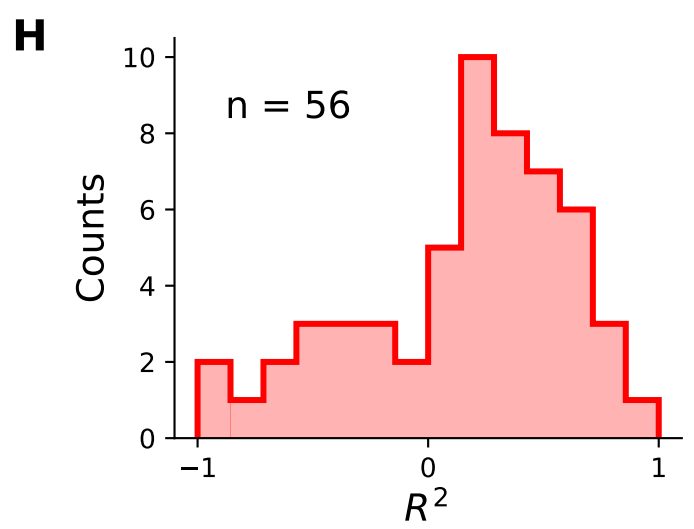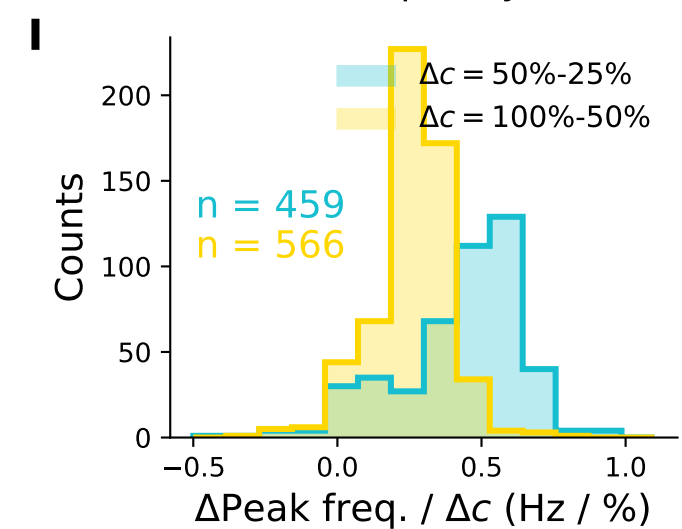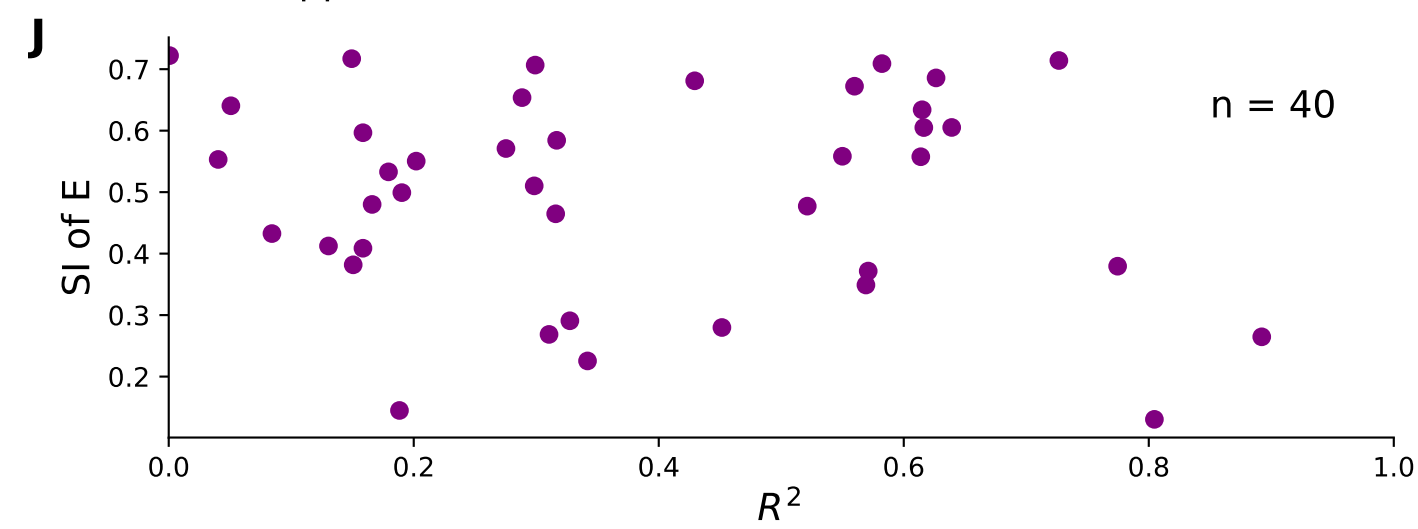

Supplement: S1 Fig — The format of the figure is exactly the same as in Fig 5 of the main text (and the reader is referred to the caption of that figure for the detailed guide). Similar to that main figure, this figure compares the locality of gamma contrast dependence in models with and without boosted intra-columnar recurrent excitatory connectivity (columnar vs. non-columnar models, respectively) across their parameter space. However, unlike those in the main figure, the sampled models here had long-range inhibitory connections. Specifically, in each sample the range of I → E and I → I connections were set to two-thirds of the randomly-sampled ranges of the E → E and E → I excitatory connections, respectively (as in the main text, the excitatory ranges, alongside other parameters, were sampled randomly over a broad range). Thus, while I connections were 33% shorter than E connections, they had long and variable ranges across samples; by contrasts, in the main Fig 5, both I → E and I → I connections had a constant range of 0.09 mm (c.f. our mini-column size of 0.4 mm), across all sampled model. As evident, e.g., from the stark contrast between the behaviour of samples in panels E vs. J (compare with the same panels in Fig 5 of main text), the qualitative difference between the columnar vs. non-columnar models (in accounting for the local contrast dependence of gamma frequency) remains unchanged in the presence of long-range inhibition; our conclusions are thus robust with respect to the assumption of very short inhibitory connections. (PDF) [file pcbi.1012190.s002.pdf]

model of Fig. 4

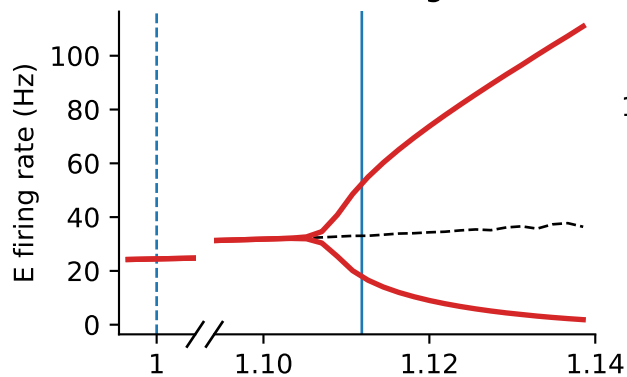

model of Fig. 6

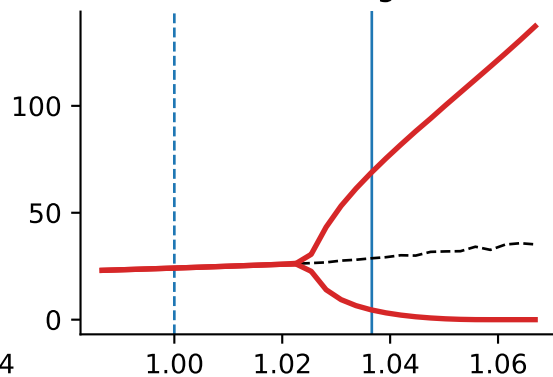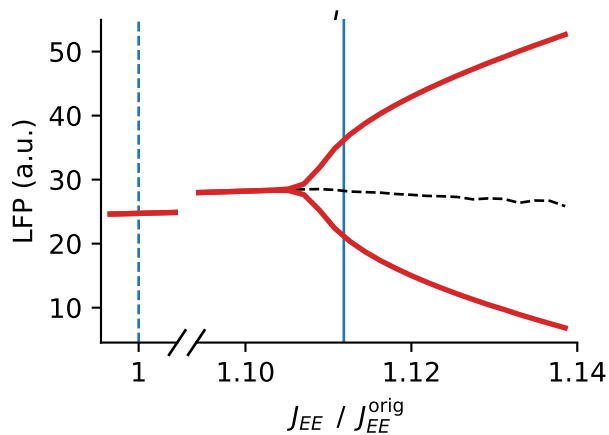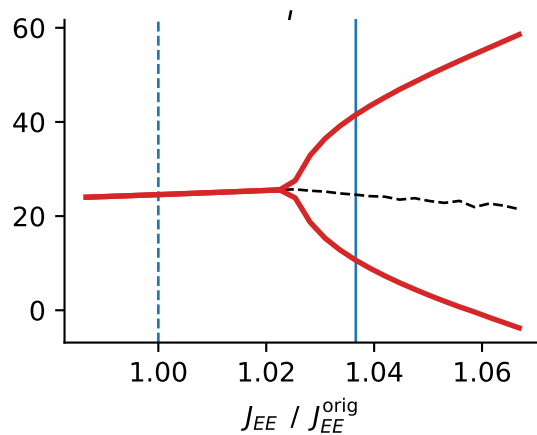

Supplement: S2 Fig — The left and right columns show the Hopf bifurcation diagrams for the two example models used in Figs 4 and 6 of the main text, respectively, when stimulated with gratings of full contrast. As the strength of recurrent excitation, JEE, is increased beyond a crticial value, the network undergoes a Hopf bifurcation, switching from a state of damped oscillations to a state with sustained oscillations. To obtain these plots, at each value of JEE, the networks were simulated without noise for long enough to reach steady state: below the Hopf bifurcation the steady state corresponds to a stable fixed point, while above the bifurcation it corresponds to a stable limit cycle (2 seconds of network dynamics were simulated, containing many tens of oscillation cycles). In the plots of the top row the lower and upper branches of the red lines show the minimum and maximum excitatory firing rates (of the E unit in the center of the model’s retinotopic grid) throughout the steady state oscillations (below the bifurcation these lines overlap, as the oscillation amplitude is zero and maximum and minimum rates are equal). In the bottom row plots, the firing rate is replaced with the LFP signal recorded at the center of the grid. The values on the x axes show the factor by which JEE was amplified over the original values in Figs 4 and 6 of the main text. The vertical solid blue lines in the left and right columns correspond to the values of JEE used in the S3 and S4 Figs, respectively (the dashed lines correspond to the original values used in the main figures). (PDF) [file pcbi.1012190.s003.pdf]

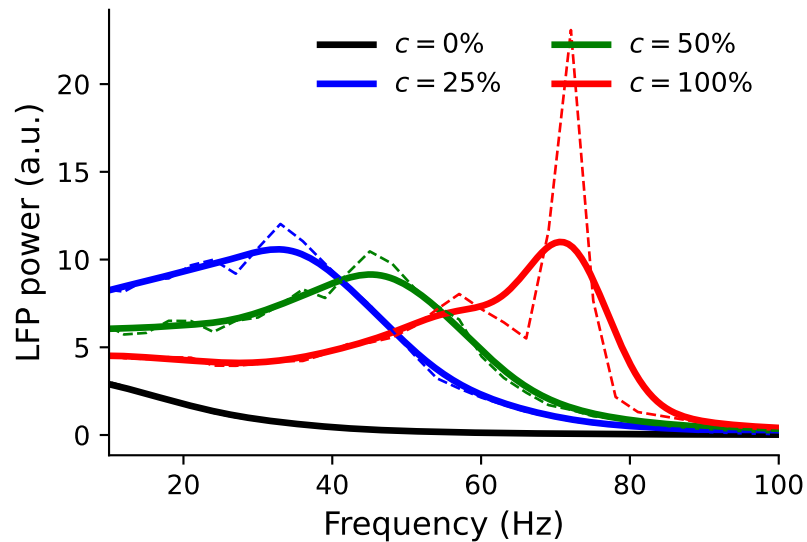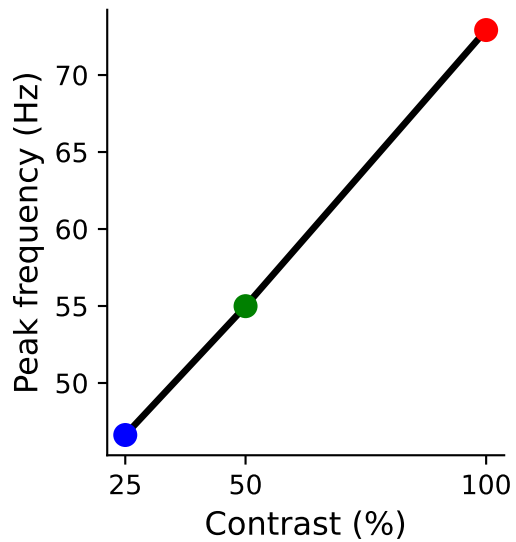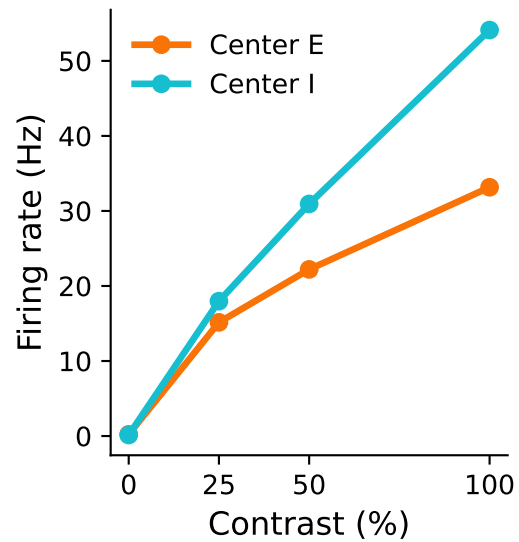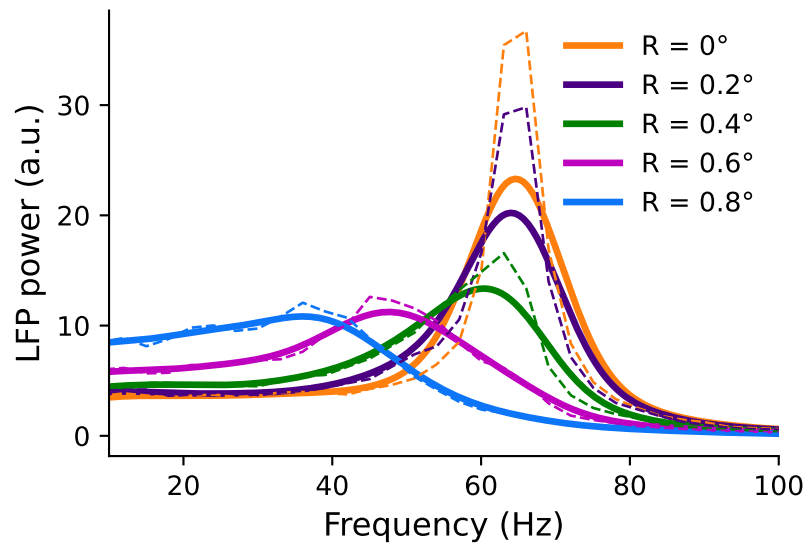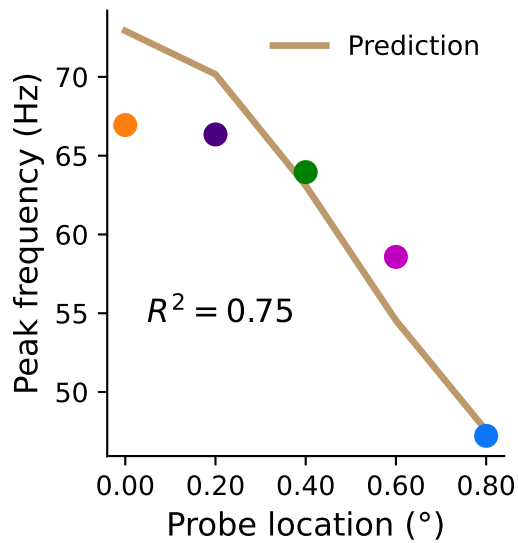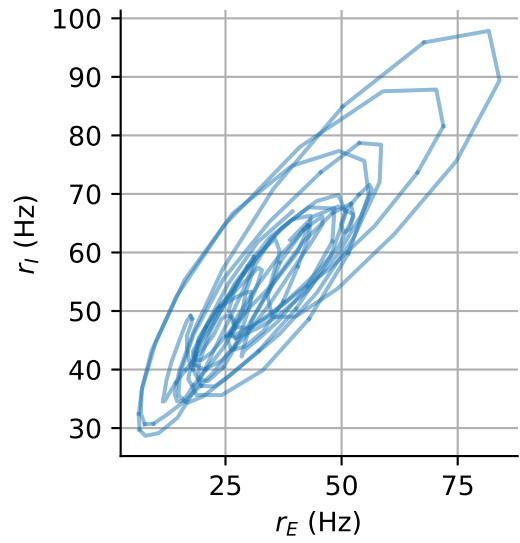

Supplement: S3 Fig — Except for the bottom right plot, the format of the rest of the figure is the same as in panels B-D and F-G of Fig 4 of the main text (and the reader is referred to the caption of that figure for the detailed guide). The parameters of the model are also the same as those in Fig 4, except for JEE which has been strengthened by a relative factor of 1.112. Unlike in Fig 4, the full stochastic dynamics of the model network were simulated (for 60 seconds, in order to allow for accurate estimation of power-spectra). In particular, the LFP power-spectra shown in the the left column plots were obtained from these simulations using the Welch periodogram method, followed by a gaussian smoothing with a σ of 5 Hz (the dashed lines show the Welch periodogram without smoothing; conclusions are not sensitive to this smoothing). The peak frequencies were obtained using the methods described in the main text from these power-spectra. The bottom-right plot shows a portion of the simulated trajectory in the plane of the E and I firing rates in the center of the retinotopic grid. These show many cycles of the noise-driven oscillations (without input noise, the same plots would have shown overlapping deterministic trajectories going around a diagonally elongated oval-shaped limit cycle). (PDF) [file pcbi.1012190.s004.pdf]

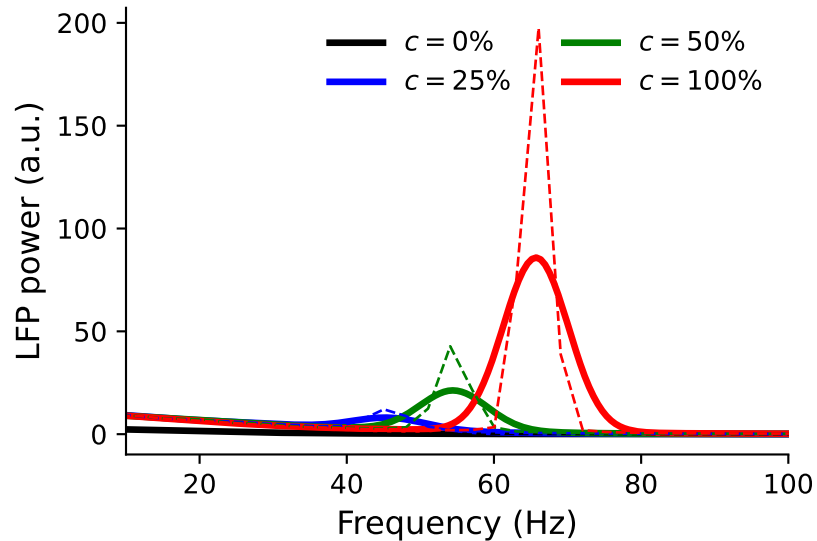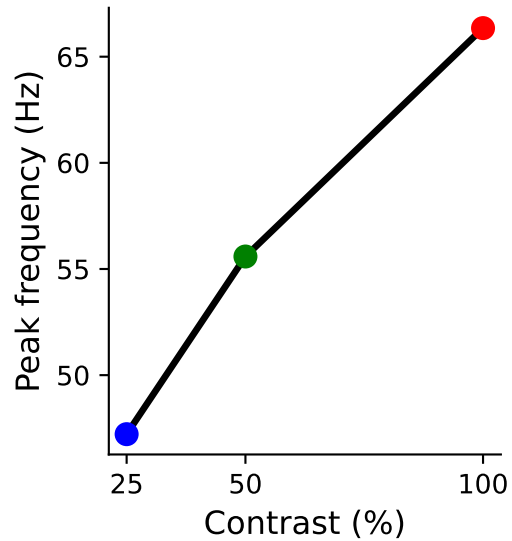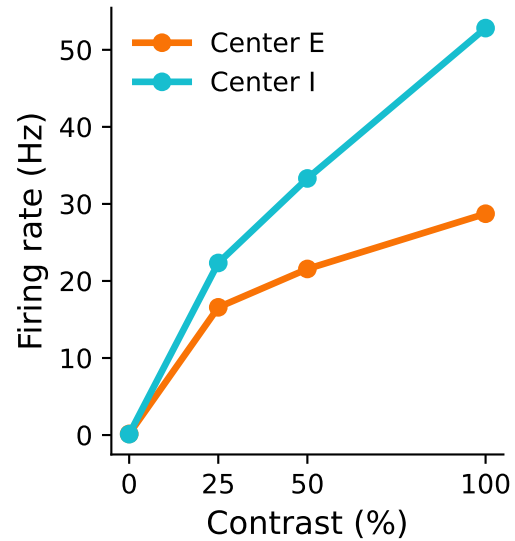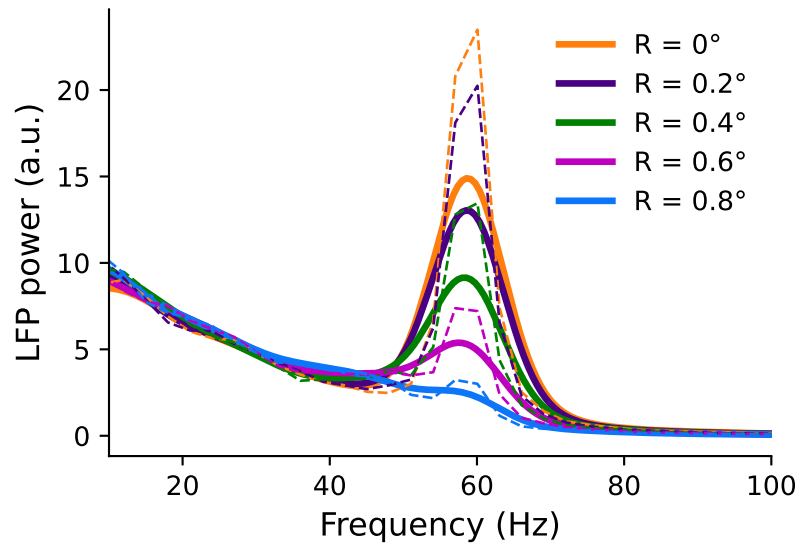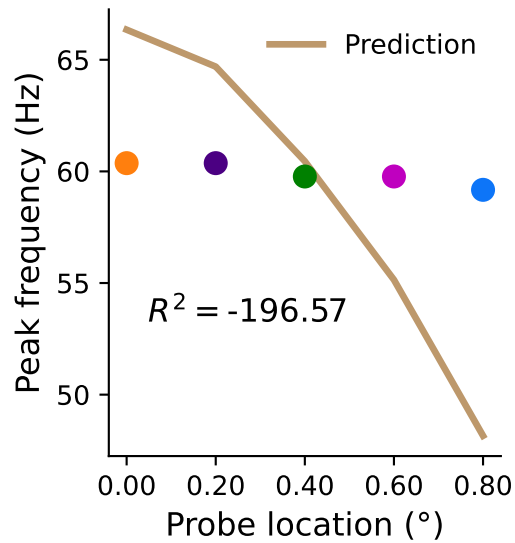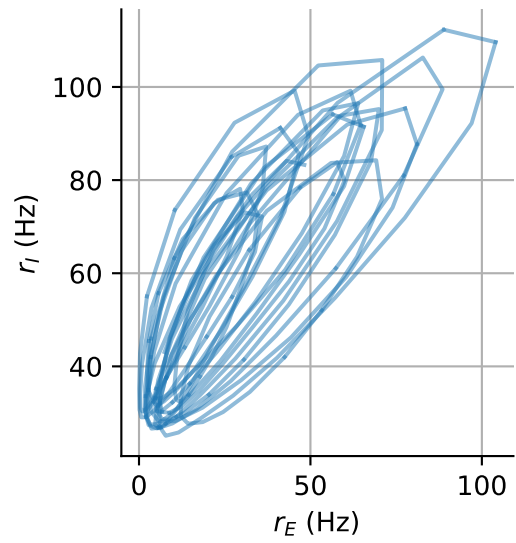

Supplement: S4 Fig — Except for the bottom-right plot, the format of the rest of the figure is the same as in Fig 6 of the main text (and the reader is referred to the caption of that figure for the detailed guide). The parameters of the model are also the same as those in Fig 6, except for JEE which has been strengthened by a relative factor of 1.037. The simulation procedure and the description of the bottom-right plot are as given in the caption of S3. (PDF) [file pcbi.1012190.s005.pdf]
