# Supplementary material for: The stabilized supralinear network accounts for the contrast dependence of visual cortical gamma oscillations
Source: PLoS Comput Biol. 2024 Jun 27;20(6):e1012190. doi: 10.1371/journal.pcbi.1012190 (PMC11236182; doi:10.1371/journal.pcbi.1012190)
Supplement: Attachment — Submitted filename: rebuttal_letter.pdf [file pcbi.1012190.s006.pdf]

Reply to reviewers

C. Holt, K. D. Miller, Y. Ahmadian

April 19, 2024

We thank both reviewers for their substantive comments and constructive criticism, which we have now addressed, leading to (we believe) a better and more complete manuscript, including four new supplementary figures. Below, we respond to their comments in detail and point out the changes made to the manuscript to address them. We have provided a "diff" PDF with the changes marked in colored text (omissions in red, additions in green). A new Supplementary Information PDF is also provided which contains the new figures. Below, reviewer comments appear in blue, and our responses to them in black.

1 Reviewer 1

Could the authors elaborate on whether their results, especially their prediction about excitatory connectivity, depend on their chosen inhibitory connections? Is their choice supported by some experimental evidence? Did the authors explore inhibitory connections with a range longer than the source column but still shorter than the excitatory range? Would the results differ qualitatively if some structure in the connectivity was included, for instance due to orientation selective columns? I think the paper would be strengthen by a discussion on this topic.

Inhibitory GABAergic connections are indeed shorter in range compared to horizontal connections made by excitatory pyramidal cells (see Lund et al. (1993), now cited in the paper, for measurements in macaque V1), whose axonal projections have long branches with the characteristic patchy arborization. However, we appreciate the value of the reviewer's point, at least from a theoretical point of view, as to how the range of inhibitory connections can affect our results and findings.

To address this, we re-ran the simulations that generated Figure 5 (involving 2000 networks with randomly sampled parameters), as follows. In the new setting we did not fix the range of inhibitory connections, σ_{EI}, and σ_{II}, to 0.09 mm (to be compared with the distance between our model's units or mini-columns, which is 0.4 mm), but rather let them be equal to two-thirds of the corresponding range for the excitatory connections (*i.e.*, $\sigma_{EI} = 2\sigma_{EE}/3$ and $\sigma_{II} = 2\sigma_{IE}/3$) in each randomly sampled network. Thus, the inhibitory connections had long ranges, albeit shorter than the excitatory ones (as suggested by the reviewer).

The results of these new simulations are summarized in Supplementary Figure 1 (which has the same format as Figure 5 of the main text). It is clear from comparison of this new figure with Figure 5 of the main text, that nothing has changed qualitatively as a result of allowing long-range inhibitory connections. Specifically, focusing on panels E and J which are the focus of the figure and speak to the tension between the requirements of producing strong surround suppression (high SI index, on the y-axis) and local contrast dependence

of gamma frequency (high R^2, on the x-axis), we see that again the model without a local excess of excitatory connections (panel J) is not capable of robustly accounting for both of these effects, while in the model with such an excess connectivity (panel E) many samples produced strong surround suppression (high SI) despite having gamma frequencies with local contrast-dependence (high R^2).

See the end of p. 12 and beginning of p. 13 (of the diff PDF) for our newly added comments on this issue, which refer to Supp. Fig. 1.

Minor comments:

1) Page 20. Left column, first paragraph.Only the fast receptors, AMPA and GABA, have timescales relevant to gamma band oscillations. These receptors have very fast rise times, which correspond to frequencies much higher than the gamma band. We therefore ignored the rise times of all receptors. This sentence sounds a bit unclear. The authors may want to mention, as they do in the main text, that they also ignored the rise times of NMDA currents since they are significantly faster than the characteristic timescales of gamma oscillations.

Thanks. To address this, we have now added two sentences (and a footnote) immediately after the sentence you quoted to clarify (1) why we have added the slow NMDA to the model, and (2) why we ignore its rise time as well. (These are marked as green at the bottom of the left column on p. 21 of the “diff PDF”.)

2) Page 20, right column. In the first paragraph, the authors clearly justify the reasons why a static input-output transfer function is a good approximation, given the synaptic dynamics they consider. However, at the end of the following paragraph, they add: However, as long as those gain filters are feature-less over the gamma band, their frequency dependence would not qualitatively affect the location of the gamma peak and its stimulus dependence. Thus we expect that the static I/O approximation will not alter our qualitative results. The authors might provide clearer insights into what they mean by 'feature-less over the gamma band'. Is this in reference to the weak dependence of responses on frequency due to the amplitude of synaptic noise and its decay time?”

Indeed, we meant “weak dependence of the neuronal I/O filter on frequency within the gamma band” —most importantly lack of features such as peaks in the filter gain therein. We added the following parenthetical remark within the part you quoted to clarify “feature-less”: “(i.e., they vary sufficiently slowly over this band of frequencies, and in particular do not have features such as peaks within this band)”. (See left column on p. 22 of the “diff PDF”).

As you have indicated, this filter is indeed expected to vary slowly when the direct input noise to the neurons are temporally correlated (colored), which in turn results in part from synaptic filtering due to the decay times of the synaptic receptors. Since this was already pointed earlier on that page, we did not point it out again.

3) Fig. 2. Caption: The statement 'Panels A-E and F-J show results for the columnar and non-columnar models, respectively' seems to be misplaced. Figure 2 only showcases panels A-E, which are related to the non-retinotopic model.

Fixed — thanks.

4) Fig. 5. Caption. Last sentence. Typo: .H and;

Fixed.

5) Methods, two lines above eq. 13. Typo: N -dimensional vector of inputs vvt
Corrected. Thank you.

6) Methods, two lines below eq. 13. Typo: and f acts element-wise. Is it f or F?
Fixed (F is correct, thanks).

2 Reviewer 2

This reviewer is however not convinced that there are clear predictions from the study that help us validate or reject the underlying mechanism of increased gamma power observed in LFPs. The study considers an E-I network which even under strong external input caused by increasing contrast, reaches a stable state in the absence of noise while exhibits transient (damped) oscillation under noise. This requires the system to be close to, but below a Hopf bifurcation without noise. The authors argue that 'Gamma oscillations do not behave like sustained oscillations, as they are not auto-coherent and their timing and duration vary stochastically, resulting in a single broad peak in the power-spectrum, with no visible higher harmonics, consistent with transient (damped) and noise driven oscillations'. However, there is a body of literature that models gamma oscillation as a noisy ISN limit cycles in stochastic models (Benayoun et al. 2010, Wallace et al. 2011, Dumont et al, 2016, Li et al, 2022, etc.). These models involve a Hopf bifurcation and capture many statistical properties of gamma oscillations. It is possible to see broad peaks in model networks with noisy limit cycles in an ISN/SSN (see above), which can vary in strength and central frequency as a function of not only network parameters, but input to the network: the imaginary part of eigenvalues vary with increasing external input, similar to this proposal. These properties appear to capture many aspects of visually evoked gamma when the neural transfer function is operating in a region of accelerating nonlinearity (Veit et al, Jadi Sejnowski). In the current study, the authors analyze the power-spectrum by applying a linearization scheme: they first find the stable point under a noise-free system and then perturb it with noise and noise-drive deviations. Then by analyzing the corresponding Fourier spectrum with Green function, they calculate the contribution of each individual eigenmode to the power spectrum characterized by ratio of LFP to the noise power spectrum, demonstrating that modes with eigenvalues having a less negative real part make stronger contribution. Whereas in the frame of noisy limit cycles in an SSN/ISN, the more positive the real part of the eigenvalue is, the greater the amplitude. It is not clear if a systematic analysis for detection of higher harmonics has been conducted on electrophysiological data (visible harmonics?). Thus it is not clear to the reviewer what makes the underlying mechanism a better choice. This is a reasonable alternate model for the observed peaks in power spectrum of LFP in visual cortex. What the reviewer would like to see additionally is a proposal for one or more experiments to test model predictions that can help support or reject this or the above mentioned alternate underlying mechanisms in a concrete way. Alternately, the authors could discuss how a Hopf bifurcation based mechanism in this retinotopic model would or would not recapitulate the experimental findings. What would possibly need to change in terms of parameters or connectivity? It is possible that either mechanisms of broad peaks (damped oscillation/noisy limit cycles in superlinear ISNs) in this retinotopic model would work. It is possible that depending on stimulus properties (large vs small, spatial frequency, etc) the underlying mechanism could switch (network on either side of Hopf bifurcation). If there are distinct model predictions for

say what would happen when you change contrast of small vs large stimuli, it is very testable in current experiments. This is important because we still dont quite understand a functional role for gamma dynamics that give these broad peaks in a clear way. They could very well be diagnostic of underlying operating regime (Ray Maunsell, 2010). The modeling will then be valuable (as something not possible experimentally) and shed light on this issue. Future studies could then explore other non-oscillatory implications of these visual cortical networks going in and out of regimes under different patterns of stimulation.

We are very thankful to the reviewer for this criticism. We have now made changes to the text, and added new figures summarizing the results of simulations of the network above the Hopf bifurcation, to address the points raised. Here we summarizes the changes and respond to the specific points raised by the reviewer.

1. First, we acknowledge that our statements in the Introduction (from which the reviewer has quoted above) on the question of whether the cortex, depending on the stimulus condition, is below or above the Hopf bifurcation were too strong and some were not justified. We agree with the reviewer that given sufficient noise, characteristics of gamma oscillations can be consistent with a regime of noisy sustained oscillations (i.e. above the Hopf bifurcation, as defined in the noise-free system). Moreover the oscillatory regime (sustained or damped oscillations) could depend on the stimulus condition. We have therefore rewritten this paragraph of the Introduction —see changes on p. 3 of the “diff PDF”.

   There, we no longer make any claims about the state of cortex relative to a hypothetical Hopf bifurcation or with respect to damped vs. sustained oscillation, but only argue (following previous studies – including some of the references mentioned by the reviewer, which we now cite there) that gamma oscillations are best modelled as noise-driven oscillations (whether above or below the Hopf bifurcation).

2. We have added a new paragraph in the (new) Linearized Approximation subsection (starting on p. 5 of the diff PDF) in which we go into some detail about the question of Hopf bifurcation, pointing out that given sufficiently strong noise, noise-driven oscillations both below and above this Hopf bifurcation can both be consistent with empirically observed characteristics of gamma (here we again cite some of the references that considered this question and were mentioned by the reviewer), and that changes in the stimulus may shift the network across this bifurcation, as defined in the noise-free network.

3. But we point out there that, by the same virtue, in the presence of noise, the Hopf bifurcation is in fact not a sharp transition but a smooth crossover, and therefore we expect that the behavior of noise-driven oscillations just below or just above the Hopf bifurcation should be similar. We add that, therefore, due to the benefits of the linearized approximation, in that it has lower computational cost (allowing for explorations of model behavior across the parameter space) and enables analytical insights, we have chosen to focus on the regime below the Hopf bifurcation in the main part of the paper.

4. However, we have now added three supplementary figures that explore the behavior of the model, and in particular the contrast-dependence of gamma frequency, above the Hopf bifurcation. In these figures we have focused on the two example retinotopic SSN’s featured in Figs 4 and 6 of the main paper. First, in Supp. Fig. 2, we map out the Hopf

bifurcation in the noise-free versions of these networks as we ramp up their recurrent excitation strength, J_{EE}. Supp. Figs. 3 and 4 have the same format as in main Figures 4 and 6 and show the behavior of gamma frequency contrast dependence and its locality in these two networks, for values of J_{EE} above the Hopf bifurcation. The gamma frequency still increases robustly with contrast, and in the Gabor stimulus condition the model with columnar structure (i.e. with a local excess in excitatory connectivity) gamma frequency is still largely controlled by the local contrast (albeit with a smaller R^2 compared to Fig. 4), while in the non-columnar model peak frequency is essentially pinned to the same value independent of recording location. We discuss these results in a new subsection of Results (starting on p. 15 of the diff PDF; also discussed on p. 20 in the Discussion). We conclude that on theoretical grounds, and given the evidence from these examples, we expect the behavior of the model and the conclusions drawn will not be sensititve to variations of parameters that shift the network across the Hopf bifurcation.

5. By virtue of the above conclusion, we believe that the phenomena that are the subject of this paper, namely the contrast dependence of gamma frequency and its local nature, are not strong or appropriate probes into (or basis for predictions about) whether the network is in a regime of connectivity resulting in sustained vs decaying oscillations (in the absence of noise). We therefore regard this important question as being rather orthogonal to the subject of (and the specific phenomenon studied in) this paper, and needing to be addressed separately. We therefore decided not to pursue or speculate on this point in the paper.

We hope that these changes (and our reasoning in point 5 above) address the criticism and the points raised by the reviewer in a satisfactory way.

Minor comments:
There is at least one typo in the method section (P20, left column 16th line: change w to w_α).

Fixed — thanks.
